# *NOS2* polymorphisms in prediction of benefit from first-line chemotherapy in metastatic colorectal cancer patients

**DOI:** 10.1371/journal.pone.0193640

**Published:** 2018-03-09

**Authors:** Marta Schirripa, Wu Zhang, Dongyun Yang, Shu Cao, Satoshi Okazaki, Fotios Loupakis, Martin D. Berger, Yan Ning, Yuji Miyamoto, Mitsukuni Suenaga, Giulia Alberti, Jordan D. West, Sara Lonardi, Taline Khoukaz, Francesca Bergamo, Francesca Battaglin, Carlotta Antoniotti, Alfredo Falcone, Sebastian Stintzing, Volker Heinemann, Heinz-Josef Lenz

**Affiliations:** 1 Medical Oncology, Norris Comprehensive Cancer Center, University of Southern California, Los Angeles, California, United States of America; 2 Department of Preventive Medicine, Norris Comprehensive Cancer Center, Keck School of Medicine, University of Southern California, Los Angeles, California, United States of America; 3 Unit of Medical Oncology 1, Department of Clinical and Experimental Oncology, Istituto Oncologico Veneto, IRCCS, Padua, Italy; 4 Department of Surgery, Oncology and Gastroenterology, University of Padova, Padua, Italy; 5 Polo Oncologico, Azienda Ospedaliero-Universitaria Pisana, Pisa, Italy; 6 Medical Oncology and Comprehensive Cancer Center University of Munich, Munich, Germany; Universita Campus Bio-Medico di Roma, ITALY

## Abstract

**Background:**

Macrophages play a crucial role in the interaction between tumor and immune system, and iNOS is known as a surrogate marker of M1 macrophages activation. The goal of the study was to investigate the role of iNOS polymorphisms as prognostic marker in mCRC patients.

**Materials and methods:**

Functional significant polymorphisms in the promoter of *INOS* gene were analyzed by PCR-based and direct DNA sequencing in 4 cohorts of patients receiving bevacizumab based first-line chemotherapy: two evaluation cohorts (TRIBE ARM A and ARM B) and two validation cohorts (FIRE 3 arm A and MOMA). The relation of the SNPs with PFS and OS was evaluated through Kaplan-Meier method and log-rank test. Subgroup analyses according to *RAS* status were preplanned.

**Results:**

In the exploratory cohort 1 (TRIBE A), patients with CCTTT *any>13repeats* (N = 57) showed improved median PFS compared with patients carrying the *≤13/≤13 repeats* variant (N = 152) (HR, 0.64; 95%CI 0.44–0.92, *p* = 0.010). Similar results were shown adopting the *>26repeats*/*≤26 repeats* (HR, 0.56; 95%CI 0.36–0.87, *p* = 0.005). In *RAS* mutant, patient with *any>13 repeats* (N = 24) had improved PFS results compared with those carrying the *≤13/≤13 repeats variant* (N = 81) (HR, 0.51; 95%CI 0.30–0.87, *p* = 30.009). Similar results were found adopting the *>26 repeats/≤26 repeats* cut off: (HR, 0.52; 95%CI 0.27–0.98, *p* = 0.035). These data were partially confirmed in the exploratory cohort 2 (TRIBE B): a better median PFS was observed in patients with *>26 repeats* vs ≤26 *repeats* (N = 205) patients. However, these data were not confirmed in the two validation cohorts.

**Conclusion:**

We failed to replicate the exploratory findings in both validation sets. The CCTTT polymorphic region of the INOS gene does not predict outcome in mCRC receiving bevacizumab based first line chemotherapy. Further investigations are needed to reveal mechanisms between tumor, immune system and chemotherapy response.

## Introduction

Significant progresses have been made over the last years in the treatment of mCRC. Currently first-line therapy of patients affected by mCRC is based on the use of a combination of cytotoxics with monoclonal antibodies either anti-VEFG (bevacizumab) or anti-EGFR (cetuximab, panitumumab). Among cytotoxics, fluoropirimidine-based treatments represent the standard of care as monotherapy, doublet combinations (FOLFOX, XELOX or FOLFIRI) or triplet combination (FOLFOXIRI) [1–6].

In the last years, immunotherapy showed promising results in solid tumors especially in lung cancer and in melanoma, demonstrating an overall survival (OS) and progression-free survival (PFS) benefit in several trials [7, 8]. The role of immunotherapy in mCRC is currently under investigation. The anti-PD1 antibodies pembrolizumab and nivolumab and the combination of nivolumab plus the anti-CTLA4 ipilimumab showed promising results in ongoing clinical trials in a subgroup of patients with microsatellite instability in advanced lines of treatment [9, 10]. Up today, many efforts are ongoing, firstly, in order to identify a role for immunotherapy in earlier lines of treatment and its possible applications in combination with chemotherapy; secondly to unveil, biomarkers leading to identify a wider range of patients possible benefitting from this strategy [11–14].

The interaction of tumor cells with stroma is closely connected with immune modulation and represents an appealing research field. In this complex mechanism macrophages play a crucial role in the balance of pro and anti tumorigenic stimuli. In particular, macrophages derived from monocyte precursors undergo specific differentiation depending on the local tissue environment [15].

The classically activated M1 macrophages are characterized by the production of high levels of pro-inflammatory cytokines, high production of reactive nitrogen and oxygen intermediates, and the promotion of Th1 responses, through the upregulation of inducible nitric oxide synthase (*NOS2* or iNOS) resulting in tumoricidal effect [15–18].

INOS is a NADPH-dependent enzyme catalyzing the production of nitric oxide (NO) from L-arginine [19] and is known as a surrogate marker of M1 macrophages activation [20, 21]. Its relation with colorectal cancer has been investigated and high levels of NOS2 expression where related to colorectal cancer progression and development and with poor prognosis [22]. A possible explanation for such phenomenon is based on the relationship between high macrophage expression and increase of VEGF production and stimulation of angiogenesis and tumor growth [23, 24]. Several polymorphisms have been identified in this gene and many studies identified a highly polymorphic pentanucleotide (CTTTT)n repeat, at position– 2.6 kb in the promoter of iNOS [25] as possible a surrogate of NOS2 expression [26, 27].

Based on the above reported considerations, we hypothesized that iNOS polymorphisms might allow clarifying mechanisms related to the interaction between immune system and CRC.and might have a prognostic role in mCRC patients receiving bevacizumab- based first line treatment in three large and modern phase II/III clinical trials.

## Materials and methods

### Patients’ population

This study investigated 4 independents cohorts of mCRC patients receiving bevacizumab based first-line treatment:

1. an evaluation cohort of 227 patients receiving FOLFIRI plus bevacizumb enrolled in the TRIBE trial (TRIBE ARM A—exploratory cohort 1),
2. an evaluation cohort of 231 patients receiving FOLFOXIRI plus bevacizumb enrolled in the TRIBE trial (TRIBE ARM B—exploratory cohort 2),
3. a validation cohort of 301 patients receiving FOLFIRI plus bevacizumb enrolled in the FIRE-3 trial (FIRE-3—validation cohort 1)
4. a validation cohort of 187 patients receiving FOLFOXIRI plus bevacizumb enrolled in the MOMA trial (MOMA–validation cohort 2).

The above-mentioned studies were carried out between 2007 and 2015 in Italy, Germany and Austria. Main inclusion criteria included: first occurrence of metastatic disease in patients with histological diagnosis of adenocarcinoma of colon or rectum. Further details on inclusion and exclusion criteria have previously been described [28–30].

In TRIBE ARM A FOLFIRI consisted of a 180 mg/m2 intravenous infusion of irinotecan followed by a 200 mg/m2 intravenous infusion of leucovorin, a 400 mg/m2 intravenous bolus of fluorouracil, and a 2400 mg/m2 continuous infusion of fluorouracil for 46 h. In TRIBE ARM B and MOMA FOLFOXIRI consisted of a 165 mg/m2 intravenous infusion of irinotecan, followed by an 85 mg/m2 intravenous infusion of oxaliplatin given concurrently with 200 mg/m2 leucovorin for 120 min, followed by a 3200 mg/m2 continuous infusion of fluorouracil for 48 h. Treatment was administered every 2 weeks for up to 12 cycles followed by maintenance treatment with fluorouracil, leucovorin until disease progression, intolerable toxicities, or patient withdrawal. Tumour assessment by CT scan was done every 8 weeks until disease progression.

In FIRE-3 trial FOLFIRI consisted of irinotecan 180 mg/m2, leucovorin 200 mg/m2, 5-fluorouracil 400 mg/m2 bolus infusion and 5-fluorouracil 2400 mg/m2 as a 48-hour continuous infusion. Treatment was administered every 2 weeks until disease progression, intolerable toxicities, or patient withdrawal. Responses were measured by contrast-enhanced computed tomography (CT) scans after 6 and 12 weeks of treatment, and every 10 weeks thereafter according to RECIST v1.0.

This study was approved by The Health Sciences Review Board (HSIRB) -The Office for the Protection of Research Subjects 3720 South Flower Street, Third Floor Los Angeles, CA 90089–0706. All patients signed an informed consent.

### Genotyping analysis

*NOS2* single-nucleotide polymorphisms (SNPs) were selected according to the following criteria:

- minor allele frequency>5% in Caucasians according to the ENSEMBL database (http://www.ensembl.org/index.html)
- citation on PubMed and possible relation with cancer [26, 27, 31].
- functional or predicted functional relevance of gene transcription or protein expression. Functional significance was predicted based on information provided by the National Institute of Environmental Health Science SNP Function Prediction, Queen’s University F-SNP, and the location of the SNP in the protein-coding region of the gene (http://snpinfo.niehs.nih.gov/snpinfo/snptag.htm).

Genomic DNA was extracted from peripheral whole blood in TRIBE and MOMA trials and from formalin-fixed paraffin-embedded (FFPE) tissues in FIRE-3 using the QIAmp Kit (Qiagen, Valencia, CA, USA) according to the manufacturer’s protocol (www.qiagen.com).

PCR-based direct DNA sequence analysis using ABI 3100A Capillary Genetic Analyzer and Sequencing Scanner v1.0 (Applied Biosystems, Waltham, MA, USA) was performed for genotyping the SNPs. The extracted DNA was amplified using the primer sets shown in Table 1, and analyzed by PCR-based direct DNA sequencing. For quality control purposes, a random selection of 10% of the samples were re-examined for each polymorphism, and the genotype concordance rate was 100%. The investigator analyzed the sequencing data using the ABI Sequencing Scanner v1.0 (Applied Biosystems, Life Technologies, Grand Island, NY, USA) and was blinded to the clinical data set.

**Table 1:** Investigated SNP characteristics.

	rs number	Location	Forward primer	Reverse Primer	Minor Allele Frequency	Recessive Allele
*CCTTT repeat*	NA	promoter region	ACCCCTGGAAGCCTACAACTGCAT	GCCACTGCACCCTAGCCTGTCTCA	NA	NA
*277AG*	rs2779248	promoter region	CTTCACCCAACCCACCTCTT	AGCTCCCTGCTGAGGAAAA	0.33	G

For the primary end point analysis, NOS2 CCTTT repeats cut-offs were defined based on the maximum chi-square method for PFS and categorized as *≤13/≤13 repeats* for patients with ≤13 repeat in each allele and *any >13 repeats* for patients with at least on allele with >13 repeats.

Exploratory analyses were conducted adopting the sum of repeats in each allele and the predefined cut off was *≤26 repeat/ >26 repeats*.

Hardy-Weinberg Equilibrium was assessed by means of Chi-square test.

### Statistical analysis

The primary aim of the study was the identification of a prognostic/predictive role for the selected SNPs (*NOS2* rs27779248 and CCTTT repeat) in mCRC patients treated with first-line chemotherapy with bevacizumab. Primary endpoint was PFS. The secondary endpoints were OS and tumor response. Table 1 shows SNPs characteristics and adopted primers.

The distribution of patient baseline clinicopathologic characteristics were compared using the Chi-square test between the four cohorts.

The impact of *NOS2* SNPS in relation with FOLFIRI plus bevacizumab treatment was evaluated in TRIBE arm A and in FIRE-3 as exploratory and validation cohorts respectively.

The impact of NOS2 SNPS in relation with the addiction of oxaliplatin was evaluated in TRIBE arm B and in MOMA as exploratory and validation cohorts respectively.

The relation of the *NOS2* SNPs with PFS and OS was evaluated through Kaplan-Meier method and log-rank test in the overall population.

OS was defined as the time from randomization to death due to any cause, PFS was defined as the time from randomization to first documented disease progression or death due to any cause. Patients were censored at the time of last follow-up if no event observed. Patients were defined as responders when achieving complete or partial response and non-responders when stable or progressive disease occurred as defined by RECIST 1.0 criteria. The relation of the *NOS2* SNPs with PFS and OS was evaluated through Kaplan-Meier method and log-rank test in the overall population.

Multivariable Cox regression model was used to assess the independent effect of two SNPs on PFS and OS when adjusting for the baseline demographic, clinical, and tumor characteristics that were associated with outcomes. Correlation of SNPs with response was estimated by the multivariable logistic regression, adjusting for the same baseline characteristics. Subgroup analyses according to *RAS* status were preplanned.

With 209 patients (163 PFS events) in the exploratory cohort 1, we would have 80% power to detect a minimum hazard ratio (HR) of 0.59 to 0.65 on PFS for a SNP with a minor allele frequency from 0.1 to 0.5 using a two-sided 0.05 level log-rank test. When applied the same model and test, we would have at least 81%, 89%, and 74% power to detect the same HR in the exploratory cohort 2 (225 patients, 161 PFS events), validation cohort 1 (280 patients, 239 PFS events), and validation cohort 2 (178 patients, 125 PFS events), respectively.

All statistical analyses were performed by SAS 9.4 (SAS Institute, Cary, NC, USA). All tests were two sided at a significant level of 0.05.

## Results

Baseline clinicopathologic characteristics in the four cohorts are summarized in Table 2. Differences in terms of age, primary tumor site, primary tumor resection, performance status, *KRAS* and *RAS* status were identified across patients enrolled in the trials (Table 2).

**Table 2:** Clinicopathological features of patients.

Characteristics	N	TRIBE arm A Exploratory cohort 1 N = 209	TRIBE arm B Exploratory cohort 2 N = 225	FIRE-3 Validation cohort 1 N = 280	MOMA Validation cohort 2 N = 178	P Value
**Sex**						0.27
Males	553	128 (61%)	136 (60%)	186 (66%)	103 (58%)	
Females	339	81 (39%)	89 (40%)	94 (34%)	75 (42%)	
**Age**						**<0.001**
≤ 65	576	151 (72%)	152 (68%)	145 (52%)	128 (72%)	
> 65	316	58 (28%)	73 (32%)	135 (48%)	50 (28%)	
**Primary tumor site**						**0.041**
Right side	263	53 (25%)	76 (34%)	71 (25%)	63 (35%)	
Left side	598	143 (68%)	138 (61%)	202 (72%)	115 (65%)	
**Number of metastases**						0.50
≤1	287	89 (43%)	95 (42%)	103 (37%)	NA	
2	255	83 (40%)	91 (40%)	81 (29%)	NA	
≥3	129	37 (18%)	39 (17%)	53 (19%)	NA	
**Liver limited disease**						0.72
Yes	285	63 (30%)	77 (34%)	92 (33%)	53 (30%)	
No	607	146 (70%)	148 (66%)	188 (67%)	125 (70%)	
**Synchronous disease**						0.22
Yes	528	172 (82%)	177 (79%)	179 (64%)	NA	
No	143	37 (18%)	48 (21%)	58 (21%)	NA	
**Primary tumor resection**						**<0.001**
Yes	570	131 (63%)	153 (68%)	245 (88%)	41 (23%)	
No	322	78 (37%)	72 (32%)	35 (13%)	137 (77%)	
**Adjuvant chemotherapy**						0.13
Yes	106	26 (12%)	29 (13%)	51 (18%)	NA	
No	608	183 (88%)	196 (87%)	229 (82%)	NA	
**Performance status**						**<0.001**
ECOG 0	677	170 (81%)	200 (89%)	153 (55%)	154 (87%)	
ECOG 1	214	38 (18%)	25 (11%)	127 (45%)	24 (13%)	
**KRAS status**						**<0.001**
Wildtype	412	88 (42%)	87 (39%)	237 (85%)	NA	
Mutant	225	83 (40%)	99 (44%)	43 (15%)	NA	
**RAS status**						**<0.001**
Wildtype	345	50 (24%)	59 (26%)	191 (68%)	45 (25%)	
Mutant	412	105 (50%)	112 (50%)	77 (28%)	118 (66%)	
**BRAF status**						0.53
Wildtype	727	160 (77%)	173 (77%)	248 (89%)	146 (82%)	
Mutant	64	11 (5%)	13 (6%)	23 (8%)	17 (10%)	

### TRIBE ARM A—Exploratory cohort 1

Associations of clinicopathologic characteristics of exploratory cohort 1 with outcome are presented in S1 Table. Median follow up was 50.6 months; median PFS and OS were respectively 9.5 months and 25.8 months. Resection of the primary tumors, ECOG performance status, and *BRAF* status were significantly associated with PFS and OS. Furthermore age, primary tumor site, time to metastases, and previous adjuvant chemotherapy significantly correlated with OS (S1 Table).

#### Genotyping results

Associations between *NOS2* SNPs and outcomes in exploratory cohort 1 are summarized in Table 3.

**Table 3:** Associations between CCTTT repeats and outcomes.

		Tumor response		Progression-free survival			Overall survival		
SNP	*N*	Yes	No	Median (95%CI), months	Univariate HR (95%CI)	Multivariable HR (95%CI)	Median (95%CI), months	Univariate HR (95%CI)	Multivariable HR (95%CI)
**Exploratory cohort 1—TRIBE arm A (FOLFIRI plus bev)**									
**NOS2 rs2779248**									
A/A	77	44(59%)	30(41%)	10.3(8.4,11.0)	1(reference)	1(reference)	26.1(21.1,33.6)	1(reference)	1(reference)
A/G	106	63(61%)	41(39%)	9.7(9.2,11.1)	1.18(0.84,1.66)	1.12(0.79,1.61)	26.0(20.8,30.9)	1.07(0.77,1.50)	0.96(0.68,1.37)
G/G	41	19(49%)	20(51%)	9.5(7.9,13.6)	0.78(0.50,1.20)	0.77(0.48,1.22)	26.1(18.4,37.8)	1.11(0.72,1.69)	1.31(0.84,2.03)
*P* value*			0.31		0.10	0.26		0.87	0.35
A/G or G/G	147	82(57%)	61(43%)	9.7(9.3,11.1)	1.03(0.75,1.42)	1.00(0.72,1.39)	26.1(20.8,30.8)	1.08(0.79,1.48)	1.05(0.76,1.46)
*P* value*			0.87		0.85	0.99		0.62	0.76
A/A or A/G	183	107(60%)	71(40%)	9.7(9.0,10.8)	1(reference)	1(reference)	26.1(22.5,30.8)	1(reference)	1(reference)
G/G	41	19(49%)	20(51%)	9.5(7.9,13.6)	0.71(0.48,1.05)	0.72(0.47,1.10)	26.1(18.4,37.8)	1.06(0.73,1.55)	1.34(0.90,1.99)
*P* value*			0.14		0.064	0.13		0.75	0.15
**NOS2** **CCTTT**									
≤13/≤13	152	81(54%)	69(46%)	9.5(8.6,10.5)	1(reference)	1(reference)	24.9(20.5,27.9)	1(reference)	1(reference)
Any >13	57	37(71%)	15(29%)	11.1(8.8,12.7)	0.64(0.44,0.92)	0.62(0.41,0.93)	30.3(20.5,42.7)	0.75(0.53,1.07)	0.80(0.54,1.19)
*P* value*			**0.049**		**0.010**	**0.021**		0.10	0.28
≤26†	175	97(56%)	76(44%)	9.5(8.6,10.5)	1(reference)	1(reference)	25.0(20.5,27.9)	1(reference)	1(reference)
>26†	34	21(72%)	8(28%)	11.1(8.8,16.6)	0.56(0.36,0.87)	0.61(0.38,0.99)	36.1(20.5,49.1)	0.70(0.45,1.07)	0.73(0.45,1.18)
*P* value*			0.20		**0.005**	**0.047**		0.097	0.20
**Exploratory cohort 2—TRIBE arm B (FOLFOXIRI plus bev)**									
≤13/≤13	172	111(67%)	55(33%)	11.7(10.1,13.0)	1(reference)	1(reference)	28.5(23.4,34.3)	1(reference)	1(reference)
Any >13	53	35(69%)	16(31%)	12.8(9.3,17.2)	0.94(0.66,1.35)	1.11(0.76,1.64)	33.4(20.6,42.0)	0.83(0.56,1.21)	0.93(0.62,1.39)
*P* value*			0.86		0.74	0.59		0.33	0.72
≤26†	205	130(66%)	67(34%)	11.3(10.1,12.5)	1(reference)	1(reference)	28.2(23.4,33.4)	1(reference)	1(reference)
>26†	20	16(80%)	4(20%)	15.6(11.1,28.8)	0.57(0.32,1.00)	0.66(0.37,1.19)	42.0(30.9,66.1)	0.50(0.26,0.95)	0.58(0.30,1.11)
*P* value*			0.33		**0.045**	0.17		**0.031**	0.10
**Validation cohort 1—FIRE-3 (FOLFIRI plus bev)**									
≤13/≤13	229	129(61%)	83(39%)	10.0(9.2,10.9)	1(reference)	1(reference)	23.7(21.3,26.5)	1(reference)	1(reference)
Any >13	51	29(60%)	19(40%)	13.2(9.8,14.9)	0.79(0.56,1.11)	0.77(0.54,1.09)	28.8(20.1,42.9)	0.75(0.51,1.11)	0.79(0.53,1.18)
*P* value*			0.59		0.16	0.14		0.15	0.25
≤26†	253	143(61%)	91(39%)	10.1(9.3,11.3)	1(reference)	1(reference)	23.7(21.5,26.7)	1(reference)	1(reference)
>26†	27	15(58%)	11(42%)	12.0(9.8,15.1)	0.79(0.52,1.21)	0.81(0.52,1.27)	31.5(18.9,43.7)	0.69(0.43,1.13)	0.74(0.44,1.24)
*P* value*			0.51		0.27	0.36		0.14	0.25
**Validation cohort 2—MOMA (FOLFOXIRI plus bev)**									
≤13/≤13	132	83(64%)	47(36%)	9.1(7.9,9.9)	1(reference)	1(reference)	24.7(19.4,30.8)	1(reference)	1(reference)
Any >13	46	28(67%)	14(33%)	10.3(7.9,10.6)	0.99(0.67,1.47)	0.84(0.56,1.27)	27.3(16.9,43.3)	0.83(0.51,1.36)	0.86(0.52,1.43)
*P* value*			0.62		0.97	0.41		0.46	0.57
≤26†	157	99(66%)	52(34%)	9.1(8.2,9.9)	1(reference)	1(reference)	24.7(19.4,30.8)	1(reference)	1(reference)
>26†	21	12(57%)	9(43%)	10.5(7.7,12.6)	0.87(0.51,1.50)	0.86(0.49,1.50)	27.3(16.9,41.2)	0.86(0.46,1.62)	1.00(0.53,1.92)
*P* value*			0.43		0.62	0.59		0.64	0.99

* P value was based on the multivariable logistic regression for tumor response, log-rank test for PFS and OS on the univariate analysis and Wald test in the multivariable Cox regression model. Multivariable models were adjusted for sex, age, performance status, primary tumor site, primary tumor resection, adjuvant chemotherapy, number of metastases, high ALP, RAS and BRAF status in the exploratory cohorts 1 and 2; adjusted for sex, age, performance status, primary tumor site, primary tumor resection, adjuvant chemotherapy, liver limited disease, RAS status and BRAF status in the validation cohort 1; adjusted for age, performance status and liver limited disease in the validation cohort 2.

^†^ Sum of (CA) repeats of two alleles.

**Fig 1:**
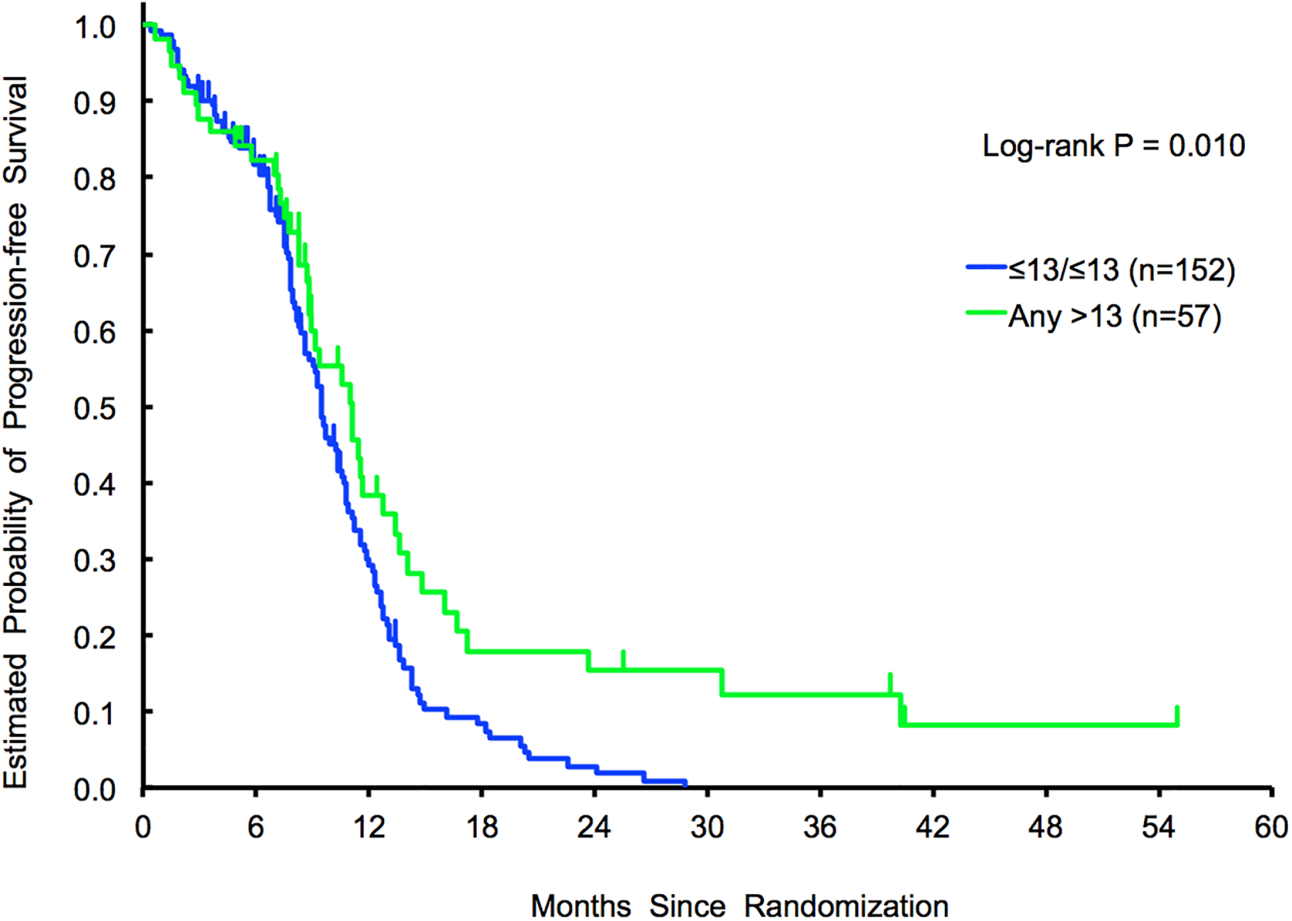
Exploratory cohort 1 (tribe—A). PFS results in *any > 13 repeats* vs *≤13/≤13 repeats*.

In the overall population, patients with CCTTT *any > 13 repeats* (N = 57) showed improved median PFS compared with patients carrying the *≤13/≤13 repeats* variants (N = 152), respectively 11.1 and 9.5 months (HR, 0.64; 95% CI 0.44–0.92, *p* = 0.010). This association remained significant in multivariable analysis (HR, 0.62, 95%, CI 0.41–0.93, *p* = 0.021) (Fig 1).

Exploratory analyses adopting the > 26 repeats (N = 34) and ≤ 26 repeats (N = 175) cut off showed similar results with a median PFS of 11.1 and 9.5 months respectively (HR, 0.56; 95% CI 0.36–0.87, *p* = 0.005). This association remained significant in multivariable analysis (HR, 0.61, 95%, CI 0.38–0.99, *p* = 0.047). No associations were identified with NOS2 rs27779248 SNP.

**Subgroup analyses:** In *RAS* mutant patients those carrying *any > 13 repeats* (N = 24) showed better median PFS compared with those with *≤13/≤13 repeats* (N = 81), 11.6 and 9.0 months respectively (HR, 0.51; 95% CI 0.30–0.87, *p* = 0.009). This association remained significant in multivariable analysis (HR, 0.40, 95%, CI 0.22–0.74, *p* = 0.003). Similar results were observed adopting the > 26 repeats (N = 15) and ≤ 26 repeats (N = 90) cut off (median PFS 11.1 and 9.2 months HR for univariate, 0.52; 95% CI 0.27–0.98, *p* = 0.035; HR for multivariate HR, 0.54, 95%, CI 0.27–1.06, *p* = 0.073). (Table 4).

**Table 4:** Associations between CCTTT repeats and outcomes in RAS mutant patients.

		Tumor response		Progression-free survival			Overall survival		
SNP	*N*	Yes	No	Median (95%CI), months	Univariate HR (95%CI)	Multivariable HR (95%CI)	Median (95%CI), months	Univariate HR (95%CI)	Multivariable HR (95%CI)
**Exploratory cohort 1— TRIBE arm A (FOLFIRI plus bev)**									
≤13/≤13	81	44(55%)	36(45%)	9.0(7.8,9.9)	1(reference)	1(reference)	22.7(19.1,27.9)	1(reference)	1(reference)
Any >13	24	15(68%)	7(32%)	11.6(8.8,14.9)	0.51(0.30,0.87)	0.40(0.22,0.74)	29.6(18.8,43.6)	0.86(0.51,1.44)	0.75(0.43,1.33)
*P* value*			0.27		**0.009**	**0.003**		0.56	0.33
≤26†	90	49(55%)	40(45%)	9.2(7.8,10.3)	1(reference)	1(reference)	23.3(19.5,28.6)	1(reference)	1(reference)
>26†	15	10(77%)	3(23%)	11.1(8.7,23.6)	0.52(0.27,0.98)	0.54(0.27,1.06)	26.1(17.8,55.0)	0.73(0.38,1.42)	0.80(0.40,1.60)
*P* value*			0.21		**0.035**	**0.073**		0.35	0.53
**Exploratory cohort 2—TRIBE arm B (FOLFOXIRI plus bev)**									
≤13/≤13	89	58(67%)	29(33%)	11.0(9.7,12.5)	1(reference)	1(reference)	26.2(20.4,31.0)	1(reference)	1(reference)
Any >13	23	15(68%)	7(32%)	13.4(9.0,23.1)	0.86(0.49,1.52)	1.12(0.60,2.09)	31.3(18.5,34.3)	0.98(0.58,1.66)	1.27(0.71,2.28)
*P* value*			0.80		0.61	0.73		0.95	0.43
≤26†	105	68(67%)	34(33%)	11.4(9.9,12.4)	1(reference)	1(reference)	25.8(20.4,30.8)	1(reference)	1(reference)
>26†	7	5(71%)	2(29%)	28.8(6.4,33.6)	0.38(0.12,1.21)	0.41(0.12,1.38)	40.0(30.9,56.3)	0.60(0.24,1.49)	0.64(0.24,1.70)
*P* value*			0.83		0.087	0.15		0.27	0.37
**Validation cohort 1—FIRE-3 (FOLFIRI plus bev)**									
≤13/≤13	63	33(55%)	27(45%)	10.1(8.3,12.7)	1(reference)	1(reference)	20.6(16.7,26.5)	1(reference)	1(reference)
Any >13	14	6(46%)	7(54%)	11.2(8.7,15.1)	0.93(0.48,1.78)	0.91(0.45,1.83)	28.4(11.2,42.9)	0.77(0.37,1.57)	0.76(0.35,1.63)
*P* value*			0.74		0.82	0.78		0.45	0.48
≤26†	68	35(54%)	30(46%)	10.1(8.5,12.5)	1(reference)	1(reference)	20.6(16.7,25.1)	1(reference)	1(reference)
>26†	9	4(50%)	4(50%)	14.4(1.9,17.6)	0.77(0.37,1.63)	0.69(0.31,1.53)	31.5(7.0,45.8)	0.59(0.25,1.39)	0.54(0.22,1.34)
*P* value*			0.88		0.49	0.36		0.21	0.19
**Validation cohort 2—MOMA (FOLFOXIRI plus bev)**									
≤13/≤13	90	61(69%)	27(31%)	9.0(7.9,10.3)	1(reference)	1(reference)	25.4(19.4,32.6)	1(reference)	1(reference)
Any >13	28	17(68%)	8(32%)	10.3(7.0,10.6)	1.23(0.74,2.03)	1.01(0.59,1.73)	27.3(16.2,40.9+)	0.91(0.50,1.66)	0.98(0.52,1.82)
*P* value*			0.94		0.41	0.96		0.76	0.94
≤26†	107	73(72%)	29(28%)	9.0(8.2,10.3)	1(reference)	1(reference)	24.7(18.9,32.6)	1(reference)	1(reference)
>26†	11	5(45%)	6(55%)	10.5(4.2,17.5)	0.89(0.41,1.93)	0.84(0.38,1.88)	27.9(10.3,37.2+)	0.77(0.33,1.80)	0.89(0.37,2.13)
*P* value*			0.074		0.76	0.67		0.54	0.80

* P value was based on the multivariable logistic regression for tumor response, log-rank test for PFS and OS on the univariate analysis and Wald test in the multivariable Cox regression model. Multivariable models were adjusted for sex, age, performance status, primary tumor site, primary tumor resection, adjuvant chemotherapy, number of metastases, high ALP, RAS and BRAF status in the exploratory cohorts 1 and 2; adjusted for sex, age, performance status, primary tumor site, primary tumor resection, adjuvant chemotherapy, liver limited disease, RAS status and BRAF status in the validation cohort 1; adjusted for age, performance status and liver limited disease in the validation cohort 2.

^†^ Sum of (CA) repeats of two alleles.

### TRIBE ARM B—Exploratory cohort 2

Patients characteristics and association with outcome for exploratory cohort 2 are presented in S2 Table. The median follow up was 46.4 months; median PFS and OS were 11.7 and 30.3 months, respectively. Time to metastases and resection of the primary tumors were significantly associated with PFS and OS. Primary tumor site, number of metastases, ECOG performance status, *BRAF* status were also significantly associated with OS (S2 Table).

#### Genotyping results

Associations between CCTTT repeats and outcomes in exploratory cohort 2 are summarized in Table 3.

**Fig 2:**
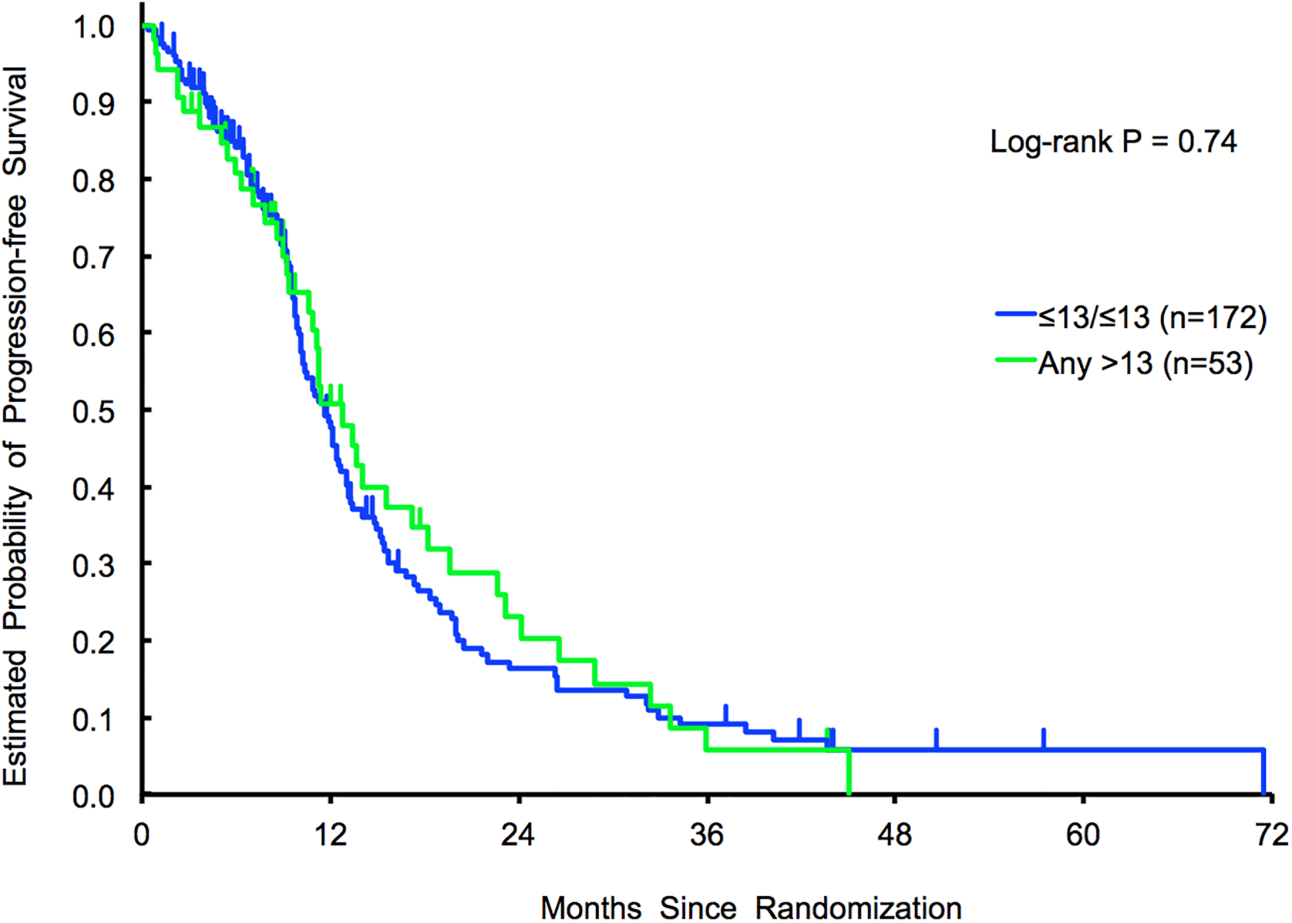
Exploratory cohort 2 (tribe—B). PFS results in *any > 13 repeats* vs *≤13/≤13 repeats*.

No association with outcome was identified adopting the *any > 13* (N = 53) *vs ≤13/≤13 repeats* (N = 172) cut off *(HR* for PFS in univariate, 0.94; 95% CI 0.66–1.35, *p* = 0.74) (Fig 2).

Median PFS for patients with *> 26 repeats* (N = 20) compared to ≤ 26 repeats (N = 205) was 15.6 versus 11.5 months, respectively (HR, 0.57; 95% CI 0.32–1.00, *p* = 0.045). This association remained significant in multivariable analysis (HR, 0.66, 95%, CI 0.37–1.19, *p* = 0.017). Moreover, patients with *> 26 repeats* had a better median OS compared with those carrying ≤ 26 repeats, 42.0 and 28.2 months respectively (HR, 0.50; 95% CI 0.26–0.95, *p* = 0.031), however such association was lost in multivariate analyses (HR, 0.58; 95% CI 0.30–1.11, *p* = 0.10).

**Subgroup analyses:** among *RAS* mutant patients no significant outcome differences in PFS were observed when adopting the *any > 13 vs ≤13/≤13 repeats* cut off (HR for univariate, 0.86; 95% CI 0.49–1.52, *p* = 0.61; HR for multivariate 1.12; 95% CI 0.60–2.09 *p* = 0.73). Patients with >26 repeats (N = 7) had a median PFS of 28.8 months compared to 11.4 months in patients with ≤ 26 repeats (N = 105), such difference was not statistically significant (HR in univariate, 0.38; 95% CI 0.12–1.21, *p* = 0.087; HR in multivariate 0.41; 95% CI 0.12–1.38, *p* = 0.15) (Table 4).

### FIRE -3—Validation cohort 1

Clinicopathologic characteristics and associations with outcome for validation cohort 1 are presented in S3 Table. The median follow up was 40.8 months; median PFS and OS were 10.3 and 24.7 months, respectively. Primary tumor site, ECOG performance status, and BRAF status were significantly associated with PFS and OS. Additionally, number of metastatic sites, liver limited disease, and time to metastasis were also significantly associated with OS (S3 Table).

#### Genotyping results

Associations between CCTTT repeats and outcomes in validation cohort 1 are summarized in Table 3.

**Fig 3:**
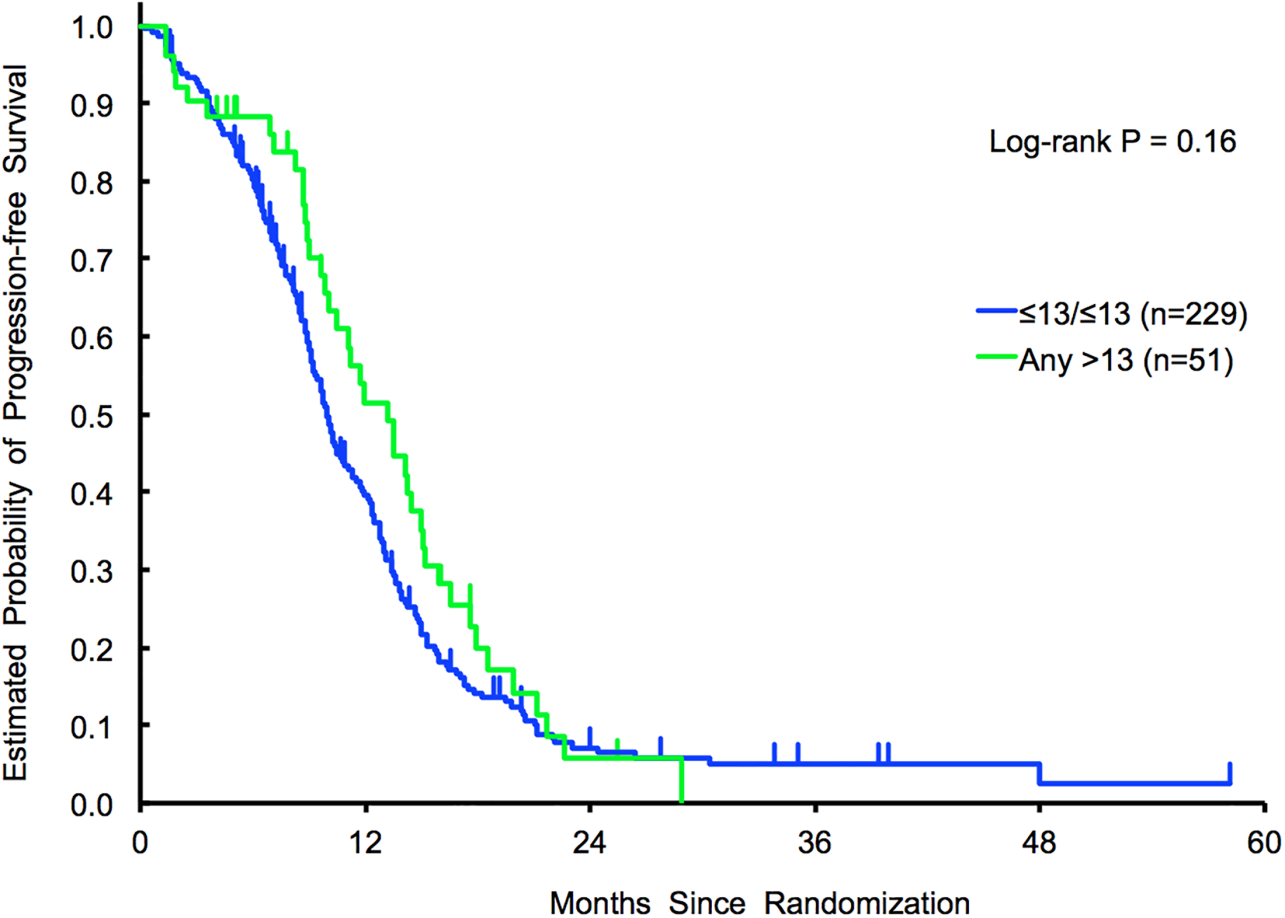
Validation cohort 1 (FIRE-3). PFS results in *any > 13 repeats* vs *≤13/≤13 repeats*.

No associations were identified in terms of outcome adopting the *any > 13* (N = 51) *vs ≤13/≤13 repeats* (N = 229) cut off (PFS: HR for univariate, 0.79; 95% CI 0.56–1.11, p = 0.16; HR for multivariate 0.77; 95% CI 0.54–1.09 p = 0.14), nor the *>26* (N = 27) *vs ≤ 26 repeats* (N = 253) cut off (PFS: HR for univariate, 0.79; 95% CI 0.52–1.21, *p* = 0.27; HR for multivariate 0.81; 95% CI 0.52–1.27 *p* = 0.36). No statistically significant outcome differences were observed among *RAS* mutant patients (N = 77) (Table 4) (Fig 3).

### MOMA—Validation cohort 2

Associations between clinical features and outcomes in validation cohort 2 are summarized in S4 Table.

The median follow up was 25.3 months, median PFS and OS were respectively 9.2 and 25.3 months. Resection of the primary tumors and ECOG performance status were significantly associated with PFS and OS; liver limited disease was also associated with OS (S4 Table).

#### Genotyping results

Associations between CCTTT repeats and outcomes in exploratory cohort 2 are summarized in Table 3.

**Fig 4:**
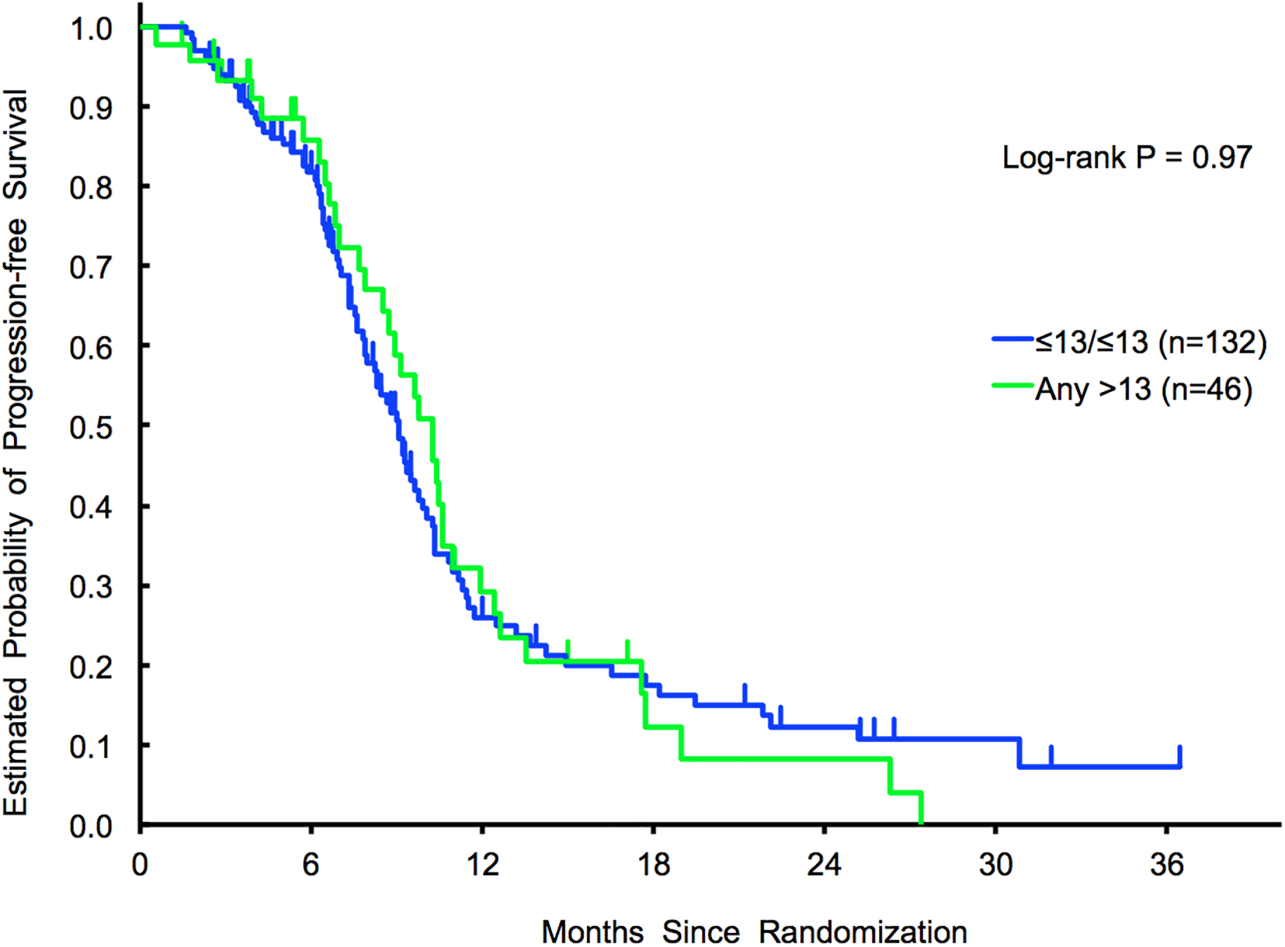
Validation cohort 2 (MOMA). PFS results in *any > 13 repeats* vs *≤13/≤13 repeats*.

No associations were identified in terms of outcome adopting the *any > 13* (N = 46) *vs ≤13/≤13 repeats* (N = 132) cut off (HR for univariate, 0.99; 95%, CI 0.67–1.47, *p* = 0.97; HR for multivariate, 0.84; 95%, CI 0.56–1.27, *p* = 0.41) nor the *>26* (N = 21) *vs ≤ 26 repeats* (N = 157) cut off (HR for univariate, 0.87; 95%, CI 0.51–1.50, *p* = 0.62; HR for multivariate, 0.86; 95%, CI 0.49–1.50, *p* = 0.59). No statistically significant outcome differences were observed among *RAS* mutant patients (N = 118) (Table 4) (Fig 4).

## Discussion

The identification of prognostic markers in mCRC patients, and in particular in the first-line treatment setting represents a crucial issue in modern clinical oncology. Prognostic factors help identifying patient subgroups with peculiar tumor phenotypes requiring personalized treatment strategies. Thanks to the recent progresses and emerging data on immunotherapeutic treatment strategies for mCRC, the interaction of immune system with colorectal cancer is becoming an appealing research field and immune-related markers are emerging as possible new prognostic or predictive factors in this setting.

For the first time a polymorphic region in the promoter of *NOS2* gene has been analyzed as surrogate marker of M1 macrophages activity and possible prognostic marker in patients receiving a first-line treatment with bevacizumab enrolled in modern, randomized phase III and II clinical trials [32–34]. We specifically selected patients receiving anti-angiogenetic drugs in modern clinical trials due to the strong association of macrophage activity and angiogenesis regulation.

The exploratory cohorts involved patients treated in the phase III TRIBE trial receiving first line treatment with FOLFIRI + bevacizumab (exploratory cohort 1) or FOLFOXIRI + bevacizumab ((exploratory cohort 2). Based on the already observed correlation of high number of NOS2 repeats with increased NOS2 expression, we hypothesized a better outcome in patients with higher number of NOS2 repeats. The role of NOS2 repeats was firstly identified in the cohort 1 but only partially confirmed in the cohort 2.

To challenge our data, we identified two validation cohorts: one treated with FOLFIRI + bevacizumab in the FIRE-3 study and the other treated with FOLFOXIRI + bevacizumab in the MOMA study. No significant clinical differences were observed among the additional 2 cohorts of patients, thus we failed to replicate the exploratory results in both validation sets. Based on the power analyses calculation, no major concerns on sample size can be raised.

In the present study we moved from a strong biological rationale [35, 36] and we selected a relatively innovative marker, since only a few studies focused on *NOS2* polymorphisms and their connection with M1 macrophage tumoral infarction [37]. Moreover nitric oxide is tightly involved in angiogenesis regulation and tumor growth [38].

As additional strengthen points we have to consider that more than 900 patients were included in the present study; study population presented comparable clinical features and was enrolled in practice changing clinical trials of first line chemotherapy for mCRC. The adoption of a rigorous study design with 2 validation cohorts increases the relevance of our findings.

As limitation points, we have to mention the lack of confirmatory preclinical data obtained from our samples, supporting the correlation between number of CCTTT repeats and iNOS expression. Unfortunately, adequate tumoral tissue was not available to solve this issue.

As possible explanation of our negative result, we have to consider that a single SNP might not catch the complexity of interactions between tumor cells and microenvironment that are regulated both by immune related factors and angiogenesis related mediators. Moreover we have to acknowledge that other sources of NO production might increase the complexity of the scenario.

In particular NO is produced not only by *NOS2* but also by the *endothelial NOS* which plays a central role in maintaining endothelial cell functional integrity. [19]. Previous studies correlated VEGF inhibition mediated by bevacizumab with a decrease in eNOS expression and thus in NO production [21].

However, despite the negative results, our findings underline the importance of investigating mechanisms regulating the interactions of tumor development, chemotherapy response and immune system in order to identify new immunotherapeutic strategies effective in patients receiving chemotherapy.

## Supporting information

S1 Table Exploratory cohort 1: Clinical characteristics and outcome results. (DOCX)

S2 Table Exploratory cohort 2: Clinical characteristics and outcome results. (DOCX)

S3 Table Validation cohort 1: Clinical characteristics and outcome results. (DOCX)

S4 Table Validation cohort 2: Clinical characteristics and outcome results. (DOCX)

## References

[1] Goldberg RM, Sargent DJ, Morton RF, Fuchs CS, Ramanathan RK, Williamson SK, et al A randomized controlled trial of fluorouracil plus leucovorin, irinotecan, and oxaliplatin combinations in patients with previously untreated metastatic colorectal cancer. Journal of clinical oncology: official journal of the American Society of Clinical Oncology. 2004;22(1):23–30. doi: 10.1200/JCO.2004.09.046 . 14665611 10.1200/JCO.2004.09.046

[2] Falcone A, Ricci S, Brunetti I, Pfanner E, Allegrini G, Barbara C, et al Phase III trial of infusional fluorouracil, leucovorin, oxaliplatin, and irinotecan (FOLFOXIRI) compared with infusional fluorouracil, leucovorin, and irinotecan (FOLFIRI) as first-line treatment for metastatic colorectal cancer: the Gruppo Oncologico Nord Ovest. Journal of clinical oncology: official journal of the American Society of Clinical Oncology. 2007;25(13):1670–6. doi: 10.1200/JCO.2006.09.0928 . 17470860 10.1200/JCO.2006.09.0928

[3] Hurwitz H, Fehrenbacher L, Novotny W, Cartwright T, Hainsworth J, Heim W, et al Bevacizumab plus irinotecan, fluorouracil, and leucovorin for metastatic colorectal cancer. N Engl J Med. 2004;350(23):2335–42. doi: 10.1056/NEJMoa032691 . 15175435 10.1056/NEJMoa032691

[4] Stathopoulos GP, Batziou C, Trafalis D, Koutantos J, Batzios S, Stathopoulos J, et al Treatment of colorectal cancer with and without bevacizumab: a phase III study. Oncology. 2010;78(5–6):376–81. doi: 10.1159/000320520 . 20798560 10.1159/000320520

[5] Cunningham D, Humblet Y, Siena S, Khayat D, Bleiberg H, Santoro A, et al Cetuximab monotherapy and cetuximab plus irinotecan in irinotecan-refractory metastatic colorectal cancer. N Engl J Med. 2004;351(4):337–45. doi: 10.1056/NEJMoa033025 . 15269313 10.1056/NEJMoa033025

[6] Jonker DJ, O’Callaghan CJ, Karapetis CS, Zalcberg JR, Tu D, Au HJ, et al Cetuximab for the treatment of colorectal cancer. N Engl J Med. 2007;357(20):2040–8. doi: 10.1056/NEJMoa071834 . 18003960 10.1056/NEJMoa071834

[7] Hellmann MD, Rizvi NA, Goldman JW, Gettinger SN, Borghaei H, Brahmer JR, et al Nivolumab plus ipilimumab as first-line treatment for advanced non-small-cell lung cancer (CheckMate 012): results of an open-label, phase 1, multicohort study. The Lancet Oncology. 2017;18(1):31–41. doi: 10.1016/S1470-2045(16)30624-6 27932067 10.1016/S1470-2045(16)30624-6 PMC5476941

[8] Larkin J, Chiarion-Sileni V, Gonzalez R, Grob JJ, Cowey CL, Lao CD, et al Combined Nivolumab and Ipilimumab or Monotherapy in Untreated Melanoma. The New England journal of medicine. 2015;373(1):23–34. doi: 10.1056/NEJMoa1504030 . 26027431 10.1056/NEJMoa1504030 PMC5698905

[9] Lynch D, Murphy A. The emerging role of immunotherapy in colorectal cancer. Annals of translational medicine. 2016;4(16):305 doi: 10.21037/atm.2016.08.29 27668225 10.21037/atm.2016.08.29 PMC5009029

[10] Andre T, Lonardi S, Wong KYM, Morse M, McDermott RS, Graham Hill A, et al Combination of nivolumab (nivo) + ipilimumab (ipi) in the treatment of patients (pts) with deficient DNA mismatch repair (dMMR)/high microsatellite instability (MSI-H) metastatic colorectal cancer (mCRC): CheckMate 142 study. Journal of clinical oncology: official journal of the American Society of Clinical Oncology. 2017;35.

[11] Herbst RS, Soria JC, Kowanetz M, Fine GD, Hamid O, Gordon MS, et al Predictive correlates of response to the anti-PD-L1 antibody MPDL3280A in cancer patients. Nature. 2014;515(7528):563–7. doi: 10.1038/nature14011 25428504 10.1038/nature14011 PMC4836193

[12] Taube JM, Klein A, Brahmer JR, Xu H, Pan X, Kim JH, et al Association of PD-1, PD-1 ligands, and other features of the tumor immune microenvironment with response to anti-PD-1 therapy. Clinical cancer research: an official journal of the American Association for Cancer Research. 2014;20(19):5064–74. doi: 10.1158/1078-0432.CCR-13-3271 24714771 10.1158/1078-0432.CCR-13-3271 PMC4185001

[13] Llosa NJ, Cruise M, Tam A, Wicks EC, Hechenbleikner EM, Taube JM, et al The vigorous immune microenvironment of microsatellite instable colon cancer is balanced by multiple counter-inhibitory checkpoints. Cancer Discov. 2015;5(1):43–51. doi: 10.1158/2159-8290.CD-14-0863 25358689 10.1158/2159-8290.CD-14-0863 PMC4293246

[14] Bupathi M, Wu C. Biomarkers for immune therapy in colorectal cancer: mismatch-repair deficiency and others. Journal of gastrointestinal oncology. 2016;7(5):713–20. doi: 10.21037/jgo.2016.07.03 27747085 10.21037/jgo.2016.07.03 PMC5056251

[15] Wiegertjes GF, Wentzel AS, Spaink HP, Elks PM, Fink IR. Polarization of immune responses in fish: The ‘macrophages first’ point of view. Mol Immunol. 2016;69:146–56. doi: 10.1016/j.molimm.2015.09.026 . 26471699 10.1016/j.molimm.2015.09.026

[16] Mills CD, Lenz LL, Harris RA. A Breakthrough: Macrophage-Directed Cancer Immunotherapy. Cancer research. 2016;76(3):513–6. doi: 10.1158/0008-5472.CAN-15-1737 26772756 10.1158/0008-5472.CAN-15-1737 PMC4738030

[17] Hagemann T, Lawrence T, McNeish I, Charles KA, Kulbe H, Thompson RG, et al "Re-educating" tumor-associated macrophages by targeting NF-kappaB. The Journal of experimental medicine. 2008;205(6):1261–8. doi: 10.1084/jem.20080108 18490490 10.1084/jem.20080108 PMC2413024

[18] Lizotte PH, Baird JR, Stevens CA, Lauer P, Green WR, Brockstedt DG, et al Attenuated Listeria monocytogenes reprograms M2-polarized tumor-associated macrophages in ovarian cancer leading to iNOS-mediated tumor cell lysis. Oncoimmunology. 2014;3:e28926 doi: 10.4161/onci.28926 25083323 10.4161/onci.28926 PMC4106169

[19] Alderton WK, Cooper CE, Knowles RG. Nitric oxide synthases: structure, function and inhibition. The Biochemical journal. 2001;357(Pt 3):593–615. 11463332 10.1042/0264-6021:3570593 PMC1221991

[20] Mosser DM, Edwards JP. Exploring the full spectrum of macrophage activation. Nat Rev Immunol. 2008;8(12):958–69. doi: 10.1038/nri2448 19029990 10.1038/nri2448 PMC2724991

[21] Takeda N, O’Dea EL, Doedens A, Kim JW, Weidemann A, Stockmann C, et al Differential activation and antagonistic function of HIF-{alpha} isoforms in macrophages are essential for NO homeostasis. Genes Dev. 2010;24(5):491–501. doi: 10.1101/gad.1881410 20194441 10.1101/gad.1881410 PMC2827844

[22] Norton SE, Dunn ET, McCall JL, Munro F, Kemp RA. Gut macrophage phenotype is dependent on the tumor microenvironment in colorectal cancer. Clinical & translational immunology. 2016;5(4):e76 doi: 10.1038/cti.2016.21 27195119 10.1038/cti.2016.21 PMC4855270

[23] Barbera-Guillem E, Nyhus JK, Wolford CC, Friece CR, Sampsel JW. Vascular endothelial growth factor secretion by tumor-infiltrating macrophages essentially supports tumor angiogenesis, and IgG immune complexes potentiate the process. Cancer Res. 2002;62(23):7042–9. . 12460925

[24] Sunakawa Y, Stintzing S, Cao S, Heinemann V, Cremolini C, Falcone A, et al Variations in genes regulating tumor-associated macrophages (TAMs) to predict outcomes of bevacizumab-based treatment in patients with metastatic colorectal cancer: results from TRIBE and FIRE3 trials. Ann Oncol. 2015;26(12):2450–6. doi: 10.1093/annonc/mdv474 26416897 10.1093/annonc/mdv474 PMC4658546

[25] Motallebipour M, Rada-Iglesias A, Jansson M, Wadelius C. The promoter of inducible nitric oxide synthase implicated in glaucoma based on genetic analysis and nuclear factor binding. Molecular vision. 2005;11:950–7. . 16288199

[26] Warpeha KM, Xu W, Liu L, Charles IG, Patterson CC, Ah-Fat F, et al Genotyping and functional analysis of a polymorphic (CCTTT)(n) repeat of NOS2A in diabetic retinopathy. FASEB journal: official publication of the Federation of American Societies for Experimental Biology. 1999;13(13):1825–32. . 10506586 10.1096/fasebj.13.13.1825

[27] Kaise M, Miwa J, Suzuki N, Mishiro S, Ohta Y, Yamasaki T, et al Inducible nitric oxide synthase gene promoter polymorphism is associated with increased gastric mRNA expression of inducible nitric oxide synthase and increased risk of gastric carcinoma. European journal of gastroenterology & hepatology. 2007;19(2):139–45. doi: 10.1097/01.meg.0000252637.11291.1d . 17272999 10.1097/01.meg.0000252637.11291.1d

[28] Loupakis F, Cremolini C, Masi G, Lonardi S, Zagonel V, Salvatore L, et al Initial therapy with FOLFOXIRI and bevacizumab for metastatic colorectal cancer. N Engl J Med. 2014;371(17):1609–18. doi: 10.1056/NEJMoa1403108 . 25337750 10.1056/NEJMoa1403108

[29] Stintzing S, Modest DP, Rossius L, Lerch MM, von Weikersthal LF, Decker T, et al FOLFIRI plus cetuximab versus FOLFIRI plus bevacizumab for metastatic colorectal cancer (FIRE-3): a post-hoc analysis of tumour dynamics in the final RAS wild-type subgroup of this randomised open-label phase 3 trial. Lancet Oncol. 2016;17(10):1426–34. doi: 10.1016/S1470-2045(16)30269-8 . 27575024 10.1016/S1470-2045(16)30269-8

[30] https://clinicaltrials.gov/ct2/show/NCT02271464.

[31] Nozoe T, Yasuda M, Honda M, Inutsuka S, Korenaga D. Immunohistochemical expression of cytokine induced nitric oxide synthase in colorectal carcinoma. Oncology reports. 2002;9(3):521–4. . 11956620

[32] Cremolini C, Loupakis F, Antoniotti C, Lupi C, Sensi E, Lonardi S, et al FOLFOXIRI plus bevacizumab versus FOLFIRI plus bevacizumab as first-line treatment of patients with metastatic colorectal cancer: updated overall survival and molecular subgroup analyses of the open-label, phase 3 TRIBE study. Lancet Oncol. 2015;16(13):1306–15. doi: 10.1016/S1470-2045(15)00122-9 . 26338525 10.1016/S1470-2045(15)00122-9

[33] Heinemann V, von Weikersthal LF, Decker T, Kiani A, Vehling-Kaiser U, Al-Batran SE, et al FOLFIRI plus cetuximab versus FOLFIRI plus bevacizumab as first-line treatment for patients with metastatic colorectal cancer (FIRE-3): a randomised, open-label, phase 3 trial. The Lancet Oncology. 2014;15(10):1065–75. doi: 10.1016/S1470-2045(14)70330-4 . 25088940 10.1016/S1470-2045(14)70330-4

[34] Falcone A, Cremolini C, Loupakis F, Lonardi S, Casagrande ME, Murgioni S, et al FOLFOXIRI plus bevacizumab (bev) followed by maintenance with bev alone or bev plus metronomic chemotherapy (metroCT) in metastatic colorectal cancer (mCRC): The phase II randomized MOMA trial. Annals of Oncology. 2016;27(suppl_6).

[35] Ropponen KM, Kellokoski JK, Lipponen PK, Eskelinen MJ, Alanne L, Alhava EM, et al Expression of inducible nitric oxide synthase in colorectal cancer and its association with prognosis. Scandinavian journal of gastroenterology. 2000;35(11):1204–11. . 11145294 10.1080/003655200750056709

[36] Edin S, Wikberg ML, Dahlin AM, Rutegard J, Oberg A, Oldenborg PA, et al The distribution of macrophages with a M1 or M2 phenotype in relation to prognosis and the molecular characteristics of colorectal cancer. PloS one. 2012;7(10):e47045 doi: 10.1371/journal.pone.0047045 23077543 10.1371/journal.pone.0047045 PMC3471949

[37] Kostourou V, Cartwright JE, Johnstone AP, Boult JK, Cullis ER, Whitley G, et al The role of tumour-derived iNOS in tumour progression and angiogenesis. Br J Cancer. 2011;104(1):83–90. doi: 10.1038/sj.bjc.6606034 21139581 10.1038/sj.bjc.6606034 PMC3039789

[38] Lala PK, Chakraborty C. Role of nitric oxide in carcinogenesis and tumour progression. Lancet Oncol. 2001;2(3):149–56. doi: 10.1016/S1470-2045(00)00256-4 . 11902565 10.1016/S1470-2045(00)00256-4

