# Supplementary material for: NOS2 polymorphisms in prediction of benefit from first-line chemotherapy in metastatic colorectal cancer patients
Source: PLoS One. 2018 Mar 9;13(3):e0193640. doi: 10.1371/journal.pone.0193640 (PMC5844536; doi:10.1371/journal.pone.0193640)
Supplement: S1 Table — (DOCX) [file pone.0193640.s001.docx]

|  |  | **Progression-free survival** | | | **Overall survival** | | | |
| --- | --- | --- | --- | --- | --- | --- | --- | --- |
|  | ***N*** | **Median (95%CI), months** | **HR (95%CI)** | ***P* value** | **Median (95%CI), months** | **HR (95%CI)** | ***P* value** |  |
| **Sex** |  |  |  | 0.58 |  |  | 0.38 |  |
| Male | 128 | 9.7(8.8,11.1) | 1(reference) |  | 25.8(20.6,31.3) | 1(reference) |  |  |
| Female | 81 | 9.5(8.6,11.1) | 0.91(0.67,1.26) |  | 24.8(20.3,30.9) | 0.87(0.63,1.19) |  |  |
| **Age** |  |  |  | 0.68 |  |  | 0.021 |  |
| ≤ 65 | 151 | 9.5(8.6,10.8) | 1(reference) |  | 27.3(22.0,33.9) | 1(reference) |  |  |
| > 65 | 58 | 10.3(9.0,11.9) | 1.07(0.77,1.50) |  | 21.3(18.6,25.8) | 1.46(1.05,2.04) |  |  |
| **Tumor site** |  |  |  | 0.072 |  |  | <0.001 |  |
| Right side | 53 | 8.8(7.1,10.3) | 1(reference) |  | 19.8(14.7,24.8) | 1(reference) |  |  |
| Left side | 143 | 10.5(9.2,11.3) | 0.73(0.51,1.03) |  | 30.9(25.1,35.9) | 0.53(0.37,0.76) |  |  |
| **Number of metastases** |  |  |  | 0.84 |  |  | 0.050 |  |
| ≤1 | 89 | 10.3(8.3,11.6) | 1(reference) |  | 30.3(24.8,35.8) | 1(reference) |  |  |
| 2 | 83 | 9.5(8.6,11.1) | 0.97(0.69,1.38) |  | 24.0(20.5,30.9) | 1.29(0.92,1.82) |  |  |
| ≥3 | 37 | 9.3(7.8,11.2) | 1.10(0.72,1.68) |  | 20.0(13.4,26.3) | 1.66(1.08,2.54) |  |  |
| **Liver limited disease** |  |  |  | 0.97 |  |  | 0.34 |  |
| Yes | 63 | 10.3(8.2,11.8) | 1(reference) |  | 28.7(20.5,34.4) | 1(reference) |  |  |
| No | 146 | 9.5(8.8,10.6) | 1.01(0.71,1.43) |  | 24.5(20.5,27.9) | 1.18(0.84,1.64) |  |  |
| **Synchronous disease** |  |  |  | 0.079 |  |  | 0.004 |  |
| Yes | 172 | 9.4(8.7,10.5) | 1(reference) |  | 24.9(19.8,27.3) | 1(reference) |  |  |
| No | 37 | 10.8(8.6,13.4) | 0.69(0.45,1.05) |  | 36.8(22.5,65.4) | 0.53(0.34,0.82) |  |  |
| **Primary resection** |  |  |  | 0.002 |  |  | 0.001 |  |
| Yes | 131 | 10.8(9.5,11.7) | 1(reference) |  | 29.8(23.9,35.8) | 1(reference) |  |  |
| No | 78 | 8.3(7.8,9.5) | 1.61(1.17,2.22) |  | 19.7(16.3,26.3) | 1.65(1.21,2.26) |  |  |
| **Adjuvant chemotherapy** |  |  |  | 0.097 |  |  | 0.010 |  |
| Yes | 26 | 11.3(8.4,14.3) | 1(reference) |  | 38.0(22.5,65.4) | 1(reference) |  |  |
| No | 183 | 9.4(8.8,10.4) | 1.52(0.92,2.52) |  | 24.8(20.0,26.9) | 1.94(1.16,3.24) |  |  |
| **Performance status** |  |  |  | 0.008 |  |  | <0.001 |  |
| ECOG 0 | 170 | 10.3(9.3,11.5) | 1(reference) |  | 28.7(25.0,33.6) | 1(reference) |  |  |
| ECOG 1 | 38 | 8.1(5.7,10.3) | 1.65(1.13,2.40) |  | 14.4(7.7,18.3) | 2.55(1.74,3.76) |  |  |
| **KRAS status** |  |  |  | 0.86 |  |  | 0.95 |  |
| Wildtype | 88 | 10.8(9.0,11.6) | 1(reference) |  | 26.9(22.0,35.9) | 1(reference) |  |  |
| Mutant | 83 | 9.2(8.6,10.8) | 0.97(0.69,1.37) |  | 24.8(19.8,32.1) | 1.01(0.72,1.42) |  |  |
| **RAS status** |  |  |  | 0.83 |  |  | 0.91 |  |
| Wildtype | 50 | 10.8(8.1,12.3) | 1(reference) |  | 24.9(15.1,35.8) | 1(reference) |  |  |
| Mutant | 105 | 9.3(8.6,10.8) | 0.96(0.65,1.41) |  | 24.0(20.0,31.6) | 0.98(0.67,1.43) |  |  |
| **BRAF status** |  |  |  | 0.003 |  |  | <0.001 |  |
| Wildtype | 160 | 10.3(9.2,11.1) | 1(reference) |  | 26.9(22.7,33.1) | 1(reference) |  |  |
| Mutant | 11 | 4.2(1.6,11.7) | 2.56(1.33,4.94) |  | 10.2(3.1,21.5) | 3.94(2.08,7.48) |  |  |

**S1 Table _ Exploratory cohort 1: Clinical characteristics and outcome results**
