# Supplementary material for: NOS2 polymorphisms in prediction of benefit from first-line chemotherapy in metastatic colorectal cancer patients
Source: PLoS One. 2018 Mar 9;13(3):e0193640. doi: 10.1371/journal.pone.0193640 (PMC5844536; doi:10.1371/journal.pone.0193640)
Supplement: S2 Table — (DOCX) [file pone.0193640.s002.docx]

|  |  | **Progression-free survival** | | | **Overall survival** | | |
| --- | --- | --- | --- | --- | --- | --- | --- |
|  | ***N*** | **Median (95%CI), months** | **HR (95%CI)** | ***P* value** | **Median (95%CI), months** | **HR (95%CI)** | ***P* value** |
| **Sex** |  |  |  | 0.97 |  |  | 0.34 |
| Male | 136 | 12.0(10.2,13.2) | 1(reference) |  | 30.3(23.4,36.0) | 1(reference) |  |
| Female | 89 | 11.3(9.8,13.5) | 0.99(0.72,1.37) |  | 30.9(21.7,35.1) | 1.16(0.85,1.60) |  |
| **Age** |  |  |  | 0.39 |  |  | 0.16 |
| ≤ 65 | 152 | 11.9(10.2,13.7) | 1(reference) |  | 30.9(25.2,37.3) | 1(reference) |  |
| > 65 | 73 | 11.7(9.1,13.0) | 1.15(0.83,1.60) |  | 27.3(20.4,33.6) | 1.26(0.91,1.75) |  |
| **Tumor site** |  |  |  | 0.67 |  |  | 0.042 |
| Right side | 76 | 10.9(8.8,12.8) | 1(reference) |  | 25.1(18.7,30.9) | 1(reference) |  |
| Left side | 138 | 12.2(10.7,13.4) | 0.93(0.67,1.30) |  | 34.3(28.1,38.2) | 0.71(0.51,0.99) |  |
| **Number of metastases** |  |  |  | 0.072 |  |  | 0.010 |
| ≤1 | 95 | 11.6(10.1,13.7) | 1(reference) |  | 34.3(26.7,42.5) | 1(reference) |  |
| 2 | 91 | 13.0(10.4,15.0) | 0.88(0.62,1.26) |  | 30.9(23.3,36.1) | 1.31(0.92,1.86) |  |
| ≥3 | 39 | 10.1(7.8,11.9) | 1.41(0.93,2.13) |  | 21.6(13.1,25.8) | 1.93(1.24,2.98) |  |
| **Liver limited disease** |  |  |  | 0.62 |  |  | 0.33 |
| Yes | 77 | 11.1(9.8,13.7) | 1(reference) |  | 33.4(23.6,39.0) | 1(reference) |  |
| No | 148 | 12.0(10.3,13.2) | 0.92(0.65,1.30) |  | 28.5(23.3,33.8) | 1.18(0.85,1.64) |  |
| **Synchronous disease** |  |  |  | 0.037 |  |  | 0.004 |
| Yes | 177 | 10.9(9.9,12.2) | 1(reference) |  | 26.9(21.6,30.9) | 1(reference) |  |
| No | 48 | 13.3(11.3,20.2) | 0.68(0.47,0.99) |  | 39.0(33.4,66.1) | 0.53(0.34,0.82) |  |
| **Primary resection** |  |  |  | 0.006 |  |  | 0.037 |
| Yes | 153 | 12.2(10.9,13.7) | 1(reference) |  | 33.4(27.3,38.0) | 1(reference) |  |
| No | 72 | 10.1(9.4,12.4) | 1.56(1.11,2.20) |  | 23.4(18.7,30.6) | 1.41(1.02,1.96) |  |
| **Adjuvant chemotherapy** |  |  |  | 0.87 |  |  | 0.11 |
| Yes | 29 | 13.3(12.1,17.3) | 1(reference) |  | 38.0(25.9,61.9) | 1(reference) |  |
| No | 196 | 11.2(10.1,12.4) | 1.04(0.67,1.60) |  | 28.5(23.4,33.6) | 1.51(0.90,2.54) |  |
| **Performance status** |  |  |  | 0.13 |  |  | 0.007 |
| ECOG 0 | 200 | 12.1(10.7,13.1) | 1(reference) |  | 31.0(26.9,35.4) | 1(reference) |  |
| ECOG 1 | 25 | 9.9(4.2,15.6) | 1.42(0.90,2.25) |  | 20.5(8.9,28.1) | 1.85(1.17,2.94) |  |
| **KRAS status** |  |  |  | 0.86 |  |  | 0.37 |
| Wildtype | 87 | 11.3(9.8,15.6) | 1(reference) |  | 34.3(23.3,40.0) | 1(reference) |  |
| Mutant | 99 | 11.9(10.3,13.0) | 0.97(0.69,1.37) |  | 28.2(21.6,31.3) | 1.17(0.83,1.64) |  |
| **RAS status** |  |  |  | 0.93 |  |  | 0.17 |
| Wildtype | 59 | 11.3(9.6,15.0) | 1(reference) |  | 37.1(23.4,42.7) | 1(reference) |  |
| Mutant | 112 | 11.7(10.3,13.0) | 1.02(0.69,1.49) |  | 27.3(21.7,31.3) | 1.30(0.89,1.90) |  |
| **BRAF status** |  |  |  | 0.11 |  |  | 0.008 |
| Wildtype | 173 | 11.9(10.4,13.3) | 1(reference) |  | 30.8(25.8,34.3) | 1(reference) |  |
| Mutant | 13 | 7.9(2.4,19.8) | 1.67(0.87,3.21) |  | 12.2(7.9,28.6) | 2.20(1.21,3.99) |  |

**S2 Table _ Exploratory cohort 2: Clinical characteristics and outcome results**
