# Supplementary material for: NOS2 polymorphisms in prediction of benefit from first-line chemotherapy in metastatic colorectal cancer patients
Source: PLoS One. 2018 Mar 9;13(3):e0193640. doi: 10.1371/journal.pone.0193640 (PMC5844536; doi:10.1371/journal.pone.0193640)
Supplement: S3 Table — (DOCX) [file pone.0193640.s003.docx]

|  |  | **Progression-free survival** | | | **Overall survival** | | | |
| --- | --- | --- | --- | --- | --- | --- | --- | --- |
|  | ***N*** | **Median (95%CI), months** | **HR (95%CI)** | ***P* value** | **Median (95%CI), months** | **HR (95%CI)** | ***P* value** |  |
| **Sex** |  |  |  | 0.071 |  |  | 0.96 |  |
| Male | 186 | 10.3(9.6,11.7) | 1(reference) |  | 24.7(20.6,27.6) | 1(reference) |  |  |
| Female | 94 | 10.1(8.9,12.4) | 1.27(0.98,1.66) |  | 24.8(21.8,30.8) | 1.01(0.75,1.36) |  |  |
| **Age** |  |  |  | 0.72 |  |  | 0.31 |  |
| ≤ 65 | 145 | 10.2(9.1,12.3) | 1(reference) |  | 22.7(18.9,28.1) | 1(reference) |  |  |
| > 65 | 135 | 10.3(9.5,11.8) | 1.05(0.81,1.35) |  | 25.4(23.1,28.4) | 0.86(0.65,1.15) |  |  |
| **Tumor site** |  |  |  | 0.031 |  |  | 0.019 |  |
| Right side | 71 | 8.8(7.5,10.5) | 1(reference) |  | 22.7(16.7,23.8) | 1(reference) |  |  |
| Left side | 202 | 11.1(9.9,12.3) | 0.73(0.54,0.98) |  | 26.7(22.3,29.6) | 0.68(0.49,0.95) |  |  |
| **Number of metastases** |  |  |  | 0.23 |  |  | 0.003 |  |
| ≤1 | 103 | 11.9(9.9,13.0) | 1(reference) |  | 27.6(21.2,36.0) | 1(reference) |  |  |
| 2 | 81 | 10.0(9.2,11.7) | 1.18(0.86,1.63) |  | 26.5(23.1,29.1) | 1.27(0.88,1.84) |  |  |
| ≥3 | 53 | 9.0(8.1,10.7) | 1.35(0.94,1.95) |  | 18.6(14.0,24.7) | 2.00(1.31,3.04) |  |  |
| **Liver limited disease** |  |  |  | 0.49 |  |  | 0.034 |  |
| Yes | 92 | 11.8(9.3,13.0) | 1(reference) |  | 26.7(21.3,34.6) | 1(reference) |  |  |
| No | 188 | 10.0(9.1,11.1) | 1.10(0.84,1.44) |  | 23.7(21.2,26.5) | 1.39(1.02,1.89) |  |  |
| **Synchronous tumor** |  |  |  | 0.13 |  |  | 0.036 |  |
| Yes | 179 | 10.4(9.6,11.8) | 1(reference) |  | 23.2(21.0,26.1) | 1(reference) |  |  |
| No | 58 | 10.3(9.3,12.9) | 0.78(0.57,1.08) |  | 29.0(23.8,43.7) | 0.67(0.46,0.98) |  |  |
| **Primary resection** |  |  |  | 0.82 |  |  | 0.052 |  |
| Yes | 245 | 10.3(9.6,11.7) | 1(reference) |  | 25.1(22.3,28.1) | 1(reference) |  |  |
| No | 35 | 10.4(7.4,13.4) | 1.05(0.70,1.56) |  | 21.2(13.8,26.5) | 1.54(0.98,2.42) |  |  |
| **Adjuvant chemotherapy** |  |  |  | 0.87 |  |  | 0.16 |  |
| Yes | 51 | 9.7(8.9,11.5) | 1(reference) |  | 27.6(21.2,42.9) | 1(reference) |  |  |
| No | 229 | 10.5(9.7,12.2) | 1.03(0.74,1.41) |  | 24.2(21.3,26.5) | 1.29(0.90,1.86) |  |  |
| **Performance status** |  |  |  | <0.001 |  |  | <0.001 |  |
| ECOG 0 | 153 | 12.4(10.4,13.4) | 1(reference) |  | 29.0(25.4,33.2) | 1(reference) |  |  |
| ECOG 1 | 127 | 9.3(8.5,10.1) | 1.57(1.21,2.02) |  | 20.6(17.6,23.1) | 1.72(1.29,2.29) |  |  |
| **KRAS status** |  |  |  | 0.37 |  |  | 0.29 |  |
| Wildtype | 237 | 10.3(9.7,11.7) | 1(reference) |  | 24.8(22.3,28.0) | 1(reference) |  |  |
| Mutant | 43 | 8.9(8.1,13.4) | 1.16(0.83,1.62) |  | 22.1(16.3,28.4) | 1.21(0.85,1.73) |  |  |
| **RAS status** |  |  |  | 0.47 |  |  | 0.16 |  |
| Wildtype | 191 | 10.3(9.6,11.7) | 1(reference) |  | 25.9(23.1,28.8) | 1(reference) |  |  |
| Mutant | 77 | 10.3(8.8,12.7) | 1.11(0.83,1.48) |  | 20.6(17.1,26.5) | 1.25(0.91,1.71) |  |  |
| **BRAF status** |  |  |  | <0.001 |  |  | <0.001 |  |
| Wildtype | 248 | 11.1(10.0,12.3) | 1(reference) |  | 26.4(23.7,28.8) | 1(reference) |  |  |
| Mutant | 23 | 6.6(4.3,7.5) | 3.29(2.10,5.17) |  | 13.5(7.5,19.5) | 2.82(1.79,4.44) |  |  |

**S3 Table _ Validation cohort 1: Clinical characteristics and outcome results**
