# Supplementary material for: NOS2 polymorphisms in prediction of benefit from first-line chemotherapy in metastatic colorectal cancer patients
Source: PLoS One. 2018 Mar 9;13(3):e0193640. doi: 10.1371/journal.pone.0193640 (PMC5844536; doi:10.1371/journal.pone.0193640)
Supplement: S4 Table — (DOCX) [file pone.0193640.s004.docx]

|  |  | **Progression-free survival** | | | **Overall survival** | | | |
| --- | --- | --- | --- | --- | --- | --- | --- | --- |
|  | ***N*** | **Median (95%CI), months** | **HR (95%CI)** | ***P* value** | **Median (95%CI), months** | **HR (95%CI)** | ***P* value** |  |
| **Sex** |  |  |  | 0.96 |  |  | 0.18 |  |
| Male | 103 | 9.1(7.8,10.2) | 1(reference) |  | 27.9(20.1,41.2) | 1(reference) |  |  |
| Female | 75 | 9.4(8.5,10.5) | 1.01(0.71,1.44) |  | 19.8(16.4,28.0) | 1.34(0.87,2.04) |  |  |
| **Age** |  |  |  | 0.070 |  |  | 0.88 |  |
| ≤ 65 | 128 | 9.1(7.9,9.9) | 1(reference) |  | 25.8(19.8,32.6) | 1(reference) |  |  |
| > 65 | 50 | 9.5(8.3,12.4) | 0.71(0.48,1.05) |  | 22.5(16.8,38.9) | 1.04(0.65,1.64) |  |  |
| **Tumor site** |  |  |  | 0.93 |  |  | 0.52 |  |
| Right side | 154 | 9.1(7.8,10.5) | 1(reference) |  | 23.0(16.8,32.6) | 1(reference) |  |  |
| Left side | 24 | 9.2(8.3,10.3) | 1.02(0.70,1.47) |  | 25.4(19.4,41.2) | 0.87(0.55,1.35) |  |  |
| **Liver limited disease** |  |  |  | 0.23 |  |  | 0.042 |  |
| Yes | 63 | 10.2(8.8,12.4) | 1(reference) |  | 40.9+(19.4,40.9+) | 1(reference) |  |  |
| No | 115 | 8.9(7.9,9.8) | 1.31(0.84,2.04) |  | 22.3(17.3,28.0) | 1.67(1.01,2.76) |  |  |
| **Primary resection** |  |  |  | <0.001 |  |  | <0.001 |  |
| Yes | 53 | 21.2+ | 1(reference) |  | 40.9+ | 1(reference) |  |  |
| No | 125 | 8.8(7.8,9.6) | 1.31(0.84,2.04) |  | 19.4(16.3,24.1) | 6.71(2.73,16.49) |  |  |
| **Performance status** |  |  |  | 0.011 |  |  | <0.001 |  |
| ECOG 0 | 41 | 9.4(8.5,10.3) | 1(reference) |  | 27.3(22.5,43.3) | 1(reference) |  |  |
| ECOG 1 | 137 | 7.3(4.3,10.3) | 1.83(1.13,2.98) |  | 16.2(6.9,22.0) | 2.80(1.60,4.90) |  |  |
| **RAS status** |  |  |  | 0.73 |  |  | 0.60 |  |
| Wildtype | 45 | 9.3(7.6,12.5) | 1(reference) |  | 28.0(17.3,43.3) | 1(reference) |  |  |
| Mutant | 118 | 9.6(8.3,10.3) | 0.93(0.61,1.41) |  | 25.4(19.5,30.8) | 1.15(0.68,1.93) |  |  |
| **BRAF status** |  |  |  | 0.45 |  |  | 0.39 |  |
| Wildtype | 146 | 9.1(8.3,10.3) | 1(reference) |  | 27.3(22.0,32.6) | 1(reference) |  |  |
| Mutant | 17 | 9.5(7.6,18.2) | 0.79(0.42,1.47) |  | 19.4(11.8,43.3) | 1.37(0.66,2.88) |  |  |

**S4 Table _ Validation cohort 2: Clinical characteristics and outcome results**
